# Carbon monoxide (CO) correlates with symptom severity, autoimmunity, and responses to probiotics treatment in a cohort of children with autism spectrum disorder (ASD): a *post-hoc* analysis of a randomized controlled trial

**DOI:** 10.1186/s12888-022-04151-3

**Published:** 2022-08-08

**Authors:** Hannah Tayla Sherman, Kevin Liu, Kenneth Kwong, Suk-Tak Chan, Alice Chukun Li, Xue-Jun Kong

**Affiliations:** 1 Athinoula A. Martinos Center for Biomedical Imaging, Massachusetts General Hospital, 149 13th Street, Charlestown, MA USA; 2 Department of Psychiatry, Beth Israel Deaconess Medical Center, Boston, MA USA

**Keywords:** Autism spectrum disorder (ASD), Carboxyhemoglobin, Autoantibodies, Microbiome, Probiotics, Inflammation, Early screening, Biomarker

## Abstract

**Background:**

Inflammation, autoimmunity, and gut-brain axis have been implicated in the pathogenesis of autism spectrum disorder (ASD). Carboxyhemoglobin (SpCO) as a non-invasive measurement of inflammation has not been studied in individuals with ASD. We conducted this post-hoc study based on our published clinical trial to explore SpCO and its association with ASD severity, autoimmunity, and response to daily *Lactobacillus plantarum* probiotic supplementation.

**Methods:**

In this study, we included 35 individuals with ASD aged 3–20 years from a previously published clinical trial of the probiotic *Lactobacillus plantarum*. Subjects were randomly assigned to receive daily *Lactobacillus plantarum* probiotic (6 × 10^10^ CFUs) or a placebo for 16 weeks. The outcomes in this analysis include Social Responsiveness Scale (SRS), Aberrant Behavior Checklist second edition (ABC-2), Clinical Global Impression (CGI) scale, SpCO measured by CO-oximetry, fecal microbiome by 16 s rRNA sequencing, blood serum inflammatory markers, autoantibodies, and oxytocin (OT) by ELISA. We performed Kendall’s correlation to examine their interrelationships and used Wilcoxon rank-sum test to compare the means of all outcomes between the two groups at baseline and 16 weeks.

**Results:**

Elevated levels of serum anti-tubulin, CaM kinase II, anti-dopamine receptor D1 (anti-D1), and SpCO were found in the majority of ASD subjects. ASD severity is correlated with SpCO (baseline, *R* = 0.38, *p* = 0.029), anti-lysoganglioside GM1 (*R* = 0.83, *p* = 0.022), anti-tubulin (*R* = 0.69, *p* = 0.042), and anti-D1 (*R* = 0.71, *p* = 0.045) in treatment group.

**Conclusions:**

The findings of the present study suggests that the easily administered and non-invasive SpCO test offers a potentially promising autoimmunity and inflammatory biomarker to screen/subgroup ASD and monitor the treatment response to probiotics. Furthermore, we propose that the associations between autoantibodies, gut microbiome profile, serum OT level, GI symptom severity, and ASD core symptom severity scores are specific to the usage of probiotic treatment in our subject cohort. Taken together, these results warrant further studies to improve ASD early diagnosis and treatment outcomes.

**Trial registration:**

ClinicalTrials.gov NCT03337035, registered November 8, 2017.

**Supplementary Information:**

The online version contains supplementary material available at 10.1186/s12888-022-04151-3.

## Background

Autism Spectrum Disorder (ASD) is an intricate neurodevelopmental disorder featuring social deficit and repetitive restrictive behaviors. According to the latest Centers for Disease Control and Prevention (CDC) release, the prevalence of ASD has rapidly increased to 1 in 54 children in the USA [1]. Inflammatory mechanism, autoimmunity, and gut-brain axis have been implicated in the etiology and pathogenesis of ASD. Anti-inflammatory agents, conventional immunotherapy and probiotics have shown promising therapeutic effects to modify the core symptoms of ASD [2–7].

Inflammatory mechanisms were widely reported to be linked with ASD and its severity. Previous studies found that inflammatory cytokines were significantly elevated in ASD subjects when compared with healthy controls [8, 9]. Meantime the brain injury and inflammatory markers, glial fibrillary acidic protein (GFAP), myelin basic protein (MBP), and S100 calcium-binding protein B (S100B), were also found to be more enriched in ASD children than healthy controls [10]; these brain injury markers and cytokine release trigger glial cell activation and inflammatory process in the brain [8, 11–14]. S100B was significantly positively correlated with the severity of problem behaviors in our previous report [6]. However, these serum markers are not readily used in clinical settings due to a lack of further validation and standardization. Furthermore, the cost of performing such assays are high for individuals.

Autoimmunity-mediated neuroinflammation and systemic inflammation were also widely reported in this field. Several serum autoantibodies have been detected in individuals with ASD, which could cause systemic inflammation and change in the tight-junctions of the blood–brain barrier (BBB), leading to permeability disruptions [15, 16]. Among the autoantibodies reported, anti-endothelial cell antibodies (AECA) were first reported in ASD individuals by Connolly AM et al. [17, 18]. Later a study reported that AECA levels correlate with ASD severity [19]. Endothelial cells, including those in the brain, maybe targeted by AECAs to cause damage resulting in vasculitis [19, 20]. Pediatric Autoimmune Neuropsychiatric Disorders Associated with Streptococcal Infections (PANDAS) was found to have significant overlap with ASD [21]. Cunningham Panel included autoantibodies Anti-Dopamine Receptor D1 (Anti-D1), Anti-Dopamine Receptor D2L (Anti-D2L), Anti-tubulin, and Anti-lysoganglioside GM1 (Anti-GM1), as well as an assay of calcium calmodulin-dependent protein kinase II (CaMK II) activity, was studied in ASD cohort and found to be related to ASD severity and response to intravenous immunoglobulin (IVIg) treatment [5]. The D1 and D2L receptors are involved in many aspects of functioning, including but not excluded to memory, motor, and impulse control. Tubulin plays a central role in maintaining neuron structure and is important for normal brain function. GM1 is involved in behavioral pathways in the brain. Finally, CaMK II plays a role in many functions but is key in learning and memory [22–24]. The antibodies could be related to autoimmune encephalitis and disease severity [14, 25–27]. These autoantibodies are also not readily applied in routine practice due to high cost and lack of further validation.

Heme oxygenase-1 (HO-1) cleaves hemoglobin to form carbon monoxide (CO), biliverdin, and iron. HO-1 is a well-studied anti-inflammatory enzyme known to be elevated in various inflammatory disorders and targeting HO-1 and CO has been proposed a therapeutic modulation for inflammation [28, 29]. Measuring carboxyhemoglobin (SpCO) level via a finger sensor of a pulse CO-oximeter is an extremely easy, quick, and non-invasive test. Recent studies of the Coronavirus disease 2019 (COVID-19) have hypothesized that oxidative stress may be a factor in causing inflammation along with an increase in CO levels [30, 31]. This relationship between SpCO level and inflammation may also be present in ASD, as oxidative stress and inflammation have been recorded, which is worthy of exploring.

Gut microbiome dysbiosis and inflammatory mechanisms were reported to have a strong correlation in the pathogenesis of ASD. We previously reported a high correlation between gut microbiome dysbiosis and cytokine dysfunction (n_ASD_ = 45; n_Healthy Control, HC_ = 41) [4]. We reported some microbiome markers and their potentials in ASD diagnosis and subgrouping, which involved a correlation of microbiome profile and autonomic dysfunction in ASD [32, 33]. Probiotics administrations were reported to have anti-inflammatory effects and therapeutic potential in both animal and human studies [34]. *L-plantarum* was found to stimulate serotonin and dopamine levels in animal studies and improve ASD behaviors in human studies [6, 35, 36]. It is known that oxytocin (OT) signaling may serve as a critical link in the gut-brain axis, which may be regulated by the supplementation of probiotics, OT was widely reported to have anti-inflammatory effects and therapeutic potential, measuring OT level is of importance to monitor inflammation, ASD core symptoms and treatment response [37, 38].

To further our understanding and clinical application of the concept of inflammation, autoimmunity, and gut-brain axis in the ASD population, we conducted this post-hoc exploratory analysis study of our recently published double-blind, randomized, placebo-controlled clinical trial with multiple related secondary outcome measurements to fill the gaps in the field [6]. We explored the easily applied SpCO measuring HO-1, and the correlations with autoantibodies including AECA and Cunningham Panel, inflammatory serum markers including cytokine interleukin-1β (IL-1β) and brain injury markers (S100B, GFAP, MBP), OT serum level, microbiome profile, GI symptom severity, ASD core symptom severity and the treatment response to probiotics *L. plantarum* from reported clinical trial. We aim to assess the potential value of using easily administered pulse CO-oximeter by comparing the serum markers for early screening, diagnosis and subgrouping ASD, and guide corresponding treatment options which have already some reported efficacy, to facilitate early intervention and improve prognosis of ASD individuals [6].

## Methods

### Study design and participants

**Fig. 1:**
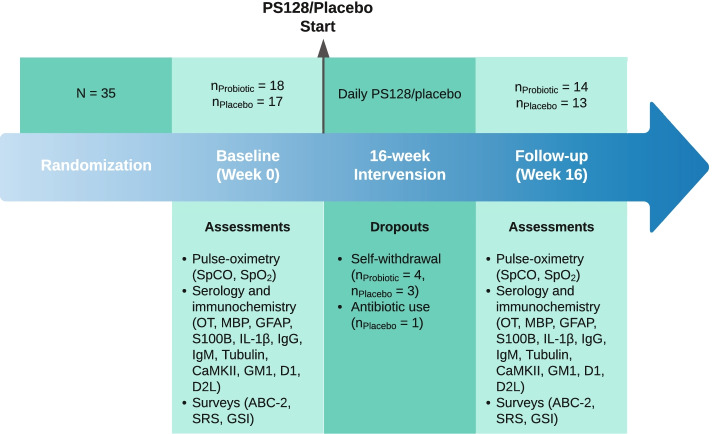
Timeline of study design, subject dropouts, and assessed variables

The original clinical trial design, protocol, randomization, blinding, participant eligibility, and intervention were well described in our previous publications [6, 33]. In this study, we conducted a *post-hoc* exploratory analysis and included all 35 subjects aged 3–25 with diagnosis of ASD from our phase 1 randomized, double blinded and placebo-controlled probiotics trial [6]. Of the 35 subjects, the probiotics group received oral probiotics PS128 (*Lactobacillus plantarum* PS128, a total of 6 × 10^10^ CFUs per day) while the placebo group received oral placebo (microcrystalline cellulose) for a total duration of 16 weeks. The study was conducted according to the guidelines of the Declaration of Helsinki. Ethical approval of the original study was issued by the Internal Review Board (IRB) of Massachusetts General Hospital (2017P001667) and this secondary use IRB (2020P004102). The original clinical trial was registered through ClinicalTrials.gov with the identifier NCT03337035 (registered November 8^th^, 2017; https://clinicaltrials.gov/ct2/show/NCT03337035). Written informed consent was obtained from either competent adult subjects, parents, or legal guardians of children and adults with cognitive impairment according to the IRB requirements. A timeline is presented in Fig. 1 to illustrate the sample size, assessed variables, and dropouts at each stage of the study.

### Outcome measures for the post-hoc analysis

#### Pulse oximetry measurements

SpCO was measured via pulse CO-oximetry which is a noninvasive technique that assesses the levels of various blood constituents, including SpCO, oxygen saturation (SpO_2_), and heart rate (HR). Measurements with the Masimo devices were taken by placing a sensor on the subject’s index or ring finger on either the right or left hands. This measurement was done in a clinical examination room over a two-minute period, where SpCO was recorded at 0 min and again at 2 min. These two records were then averaged for analysis. This measurement was collected during the primary trial but not included in previous publication. Based on previous studies of SpCO levels in cigarette smokers and non-smokers, non-smokers have been found to have SpCO values of < 1.5% [39, 40]; as a result, a percentage of ≥ 2% is denoted as positive while a measurement of < 2% is denoted as negative.

#### Blood sample collection and circulating serum biomarker measurements

**Fig. 2:**
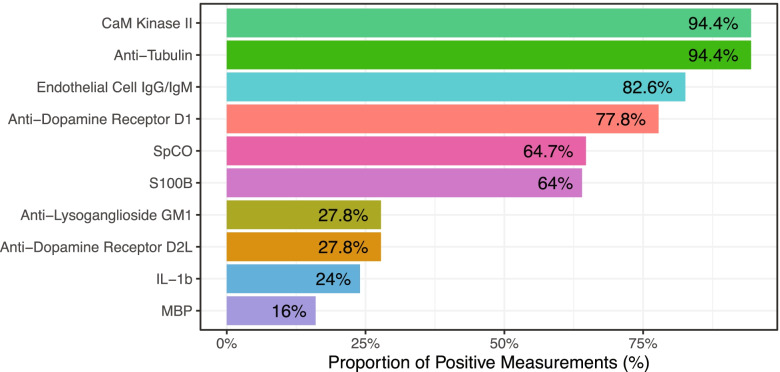
Overview of proportion of participants with detected positive SpCO and serum inflammatory markers at baseline. For SpCO, a measured percentage of ≥ 2 is denoted as “positive”; for titers of anti-Dopamine Receptor D1, D2L, anti-Lysoganglioside GM1, anti-Tubulin, and activity of CaM Kinase II of baseline, normal levels are denoted as “negative” and both borderline and elevated levels are denoted as “positive;” all remaining indices are denoted based on the absence (“negative”) or presence at detectable levels (“positive”). GFAP and OT were not included due to lack of reference for the determination of cutoff values

Blood drawn and the serum processed during the original trial week 0 and week 16, was used to measure AECAs by an ELISA kit manufactured by R&D Systems Inc. (Minneapolis, MN, USA) following the manufacturer-supplied protocol, and sent to Moleculera labs (Oklahoma City, OK) for the Cunningham Panel measurement following their instruction. These two blood measurements were not included in our previous publication. In this study, we denote positive results for CaMKII activity, anti-Tubulin, anti-D1, anti-GM1, and anti-D2L using cutoffs cited by the testing facility (Cunningham Panel, Moleculera Labs) [41, 42]. In brief, positive titer result cutoffs are determined based on the mean titer results of the healthy study population from the study conducted by Chain et al., such that anti-D1 titers with values of 2000 or higher, anti-D2L with values of 8000 or higher, anti-GM1 with values of 320 or higher, and anti-tubulin with values of 1000 or higher are considered positive results. Similarly, a positive result is denoted for CaMKII activation based on its activity with values of ≥ 130% above the basal mean activation rate [42]. Circulating serum OT, MBP, GFAP, S100B, IL-1β measurements were described in our previous publication and designations of positive and negative cutoffs relevant to the current Fig. 2 for S100B, MBP, and IL-1β are determined based on whether detectable levels were identified in our subject population [6].

#### Other outcome measurements included in this post-hoc analysis

GI symptom severity assessments by the validated GSI and the Bristol Stool Chart [43]; Social Responsiveness Scale (SRS), Aberrant Behavior Checklist second edition (ABC-2) [44, 45], Clinical Global Impression (CGI) [46], Stool Sample Processing, DNA extraction and sequencing analysis were well described in our previous publication [6].

### Bioinformatics processing and statistical analysis

The *post-hoc* analyses of a dataset generated from a double-blinded, placebo-controlled probiotic clinical trial was performed for the present study. Fecal 16S sequence reads were processed through Biobakery Workflows (v0.13.2) with default parameters using the VSEARCH-based method [47]. Assignment of OTU taxonomies were done using the Greengenes database (v13.8) with 97% sequence similarity [48]. The resulting reads were filtered using a prevalence threshold of 0.0001 and an occurrence threshold of 10%. Subsequently, the abundance data was transformed into relative abundances for downstream analysis. Linear discriminant analysis effect size (LEfSe) was used to evaluate differentially abundant microbiota relative abundances both at baseline and post-treatment using a one-against-all strategy for multi-class analysis with statistical significance considered at α = 0.05 and a threshold for the logarithmic LDA score of 2.0 [49]. The 16 s fecal microbiome sequencing data presented in this study are openly available in the Sequence Read Archive (SRA) database of The National Center for Biotechnology Information at https://www.ncbi.nlm.nih.gov/bioproject/PRJNA675093, with accession number PRJNA675093. For assessment of correlations by placebo and probiotic treatment group effects, the absolute change between baseline (0-weeks) and post-probiotic intervention (16-weeks) were calculated for pulse oximetry, and serum inflammatory markers features. Serum autoantibody titers were log2-transformed and correlational analysis was conducted using Kendall’s rank correlation. Adjustment for multiple comparisons was not performed as each test was conducted according to pre-specified hypotheses. A table of all assessed correlations are provided as an additional file [see Additional file 1].

## Results

We conducted exploratory analysis based on a cohort of 35 children with ASD aged 10.26 ± 4.78 years, which consisted of 26 males and 9 females. The maternal age at childbirth is 32.96 ± 4.9 years. Each participant was assessed for their GI symptom severity via the GSI, which suggested scores of 2.86 ± 1.77. Furthermore, the severity of ASD symptoms were assessed via the ABC-2, SRS, and CGI. The ABC-2 yielded total T scores of 275.06 ± 32.29, the SRS gave a total score of 113.8 ± 36.96, and the CGI-S gave scores of 5.11 ± 1.02. Detailed participant demographics and characteristics are summarized in Table 1. The 35 subjects were randomized assigned into two groups: probiotics group and placebo group. There were no significant differences between two groups in term of age, sex, ASD severity, and GSI as reported [6].

**Table 1:** Summary of participant demographics and clinical characteristics

Demographic Feature	All (*n* = 35)
Age (years)	10.26 ± 4.78
Sex (n)	
Male	26
Female	9
Maternal age	32.96 ± 4.9
GSI Index	2.86 ± 1.77
ABC-2 Total Score (T)	275.06 ± 32.29
SRS Total Score	113.8 ± 36.96
CGI-S	5.11 ± 1.02

We summarized the baseline levels of SpCO, serum autoantibodies, and serum inflammatory markers in this cohort. Based on such results, we found that the positive rate of the respective measurements are common and widely distributed (Fig. 2). Specifically, CaM kinase II and anti-tubulin are among the highest at 94.4% of subjects with elevated titers, followed by AECA with 82.6% of subjects with positive measurements, anti-dopamine receptor D1 (77.8%), SpCO (64.7%), S100B (64%), and anti-lysoganglioside GM1, and anti-dopamine receptor D2L both with 27.8% of subjects with elevated titers, IL-1β (24%), MBP (16%). Of note, GFAP and OT were not included due to lack of reference for the determination of cutoff values.

**Fig. 3:**
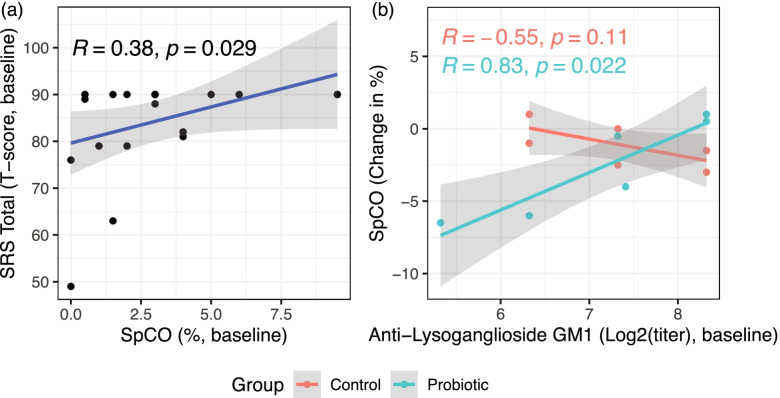
Correlations between SpCO, ASD socio-behavioral severity, and autoantibody titers at baseline and post-intervention with either probiotics (blue) or placebo (red). Shown correlations are based on Kendall’s correlation using a significance cutoff of α = 0.05. **a** SRS Total T-scores are positively correlated with SpCO for all subjects at baseline. **b** The absolute change in SpCO between weeks-0 and -16 is positively correlated with anti-lysoganglioside GM1 log-titers in those receiving the probiotic treatment

We found that the baseline SpCO was positively correlated with baseline SRS total scores (Fig. 3A, R = 0.38, *P* < 0.05), while the absolute change in SpCO post-intervention by treatment group was found to be positively correlated with baseline titers of anti-lysoganglioside GM1 among subjects receiving the active probiotic but not among those receiving the placebo control (Fig. 3B, *R*_*Probiotic*_ = 0.83, *P*_*Probiotic*_ < 0.05), which suggests that subjects with lower baseline anti-GM1 titers show a trend of decreasing SpCO post-probiotic intervention.

**Fig. 4:**
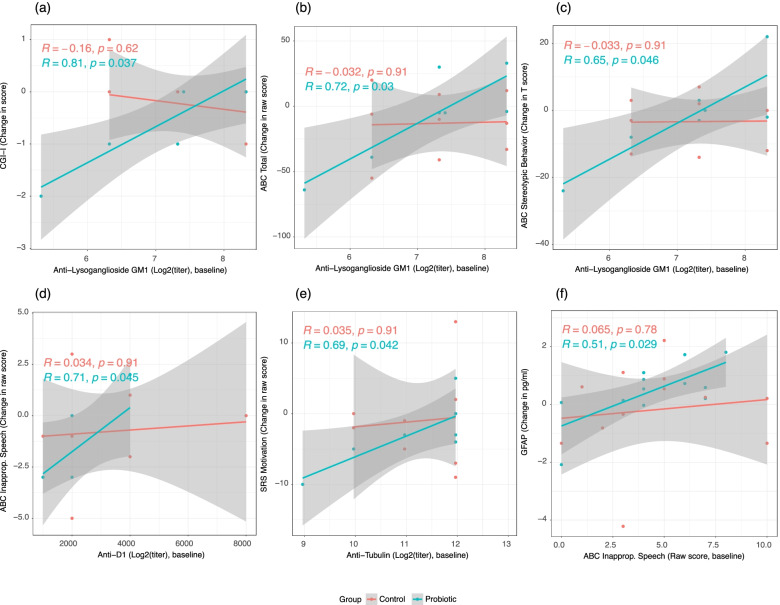
Correlations between serum autoantibody titers, inflammatory markers, and ASD severity scores, at baseline and post-intervention. Shown correlations are based on Kendall’s correlation using a significance cutoff of α = 0.05

The absolute change in CGI-I, ABC-2 total score and ABC-2 stereotypic behavior sub-score between 0-weeks and 16-weeks were found to be positively correlated with baseline titers of anti-lysoganglioside GM1 in the probiotics-treated group but not in the placebo-treated group (Fig. 4A-C, *P*_*Probiotic*_ < 0.05). Furthermore, the change in ABC-2 inappropriate speech sub-score was found to be positively correlated with baseline titers of anti-dopamine receptor D1 in the probiotics-treated group but not in the placebo-treated group (Fig. 4D, *P*_*Probiotic*_ < 0.05). Similarly, the change in SRS motivation sub-score was found to be positively correlated with baseline titers of anti-tubulin among the probiotics-treated group but was not observed in the placebo-treated group (Fig. 4E, *P*_*Probiotic*_ < 0.05). Lastly, the change in serum GFAP concentration was found to be positively correlated with baseline scores of ABC-2 inappropriate speech sub-score among subjects receiving the active probiotic but not among those receiving the placebo control (Fig. 4F, *P*_*Probiotic*_ < 0.05).

**Fig. 5:**
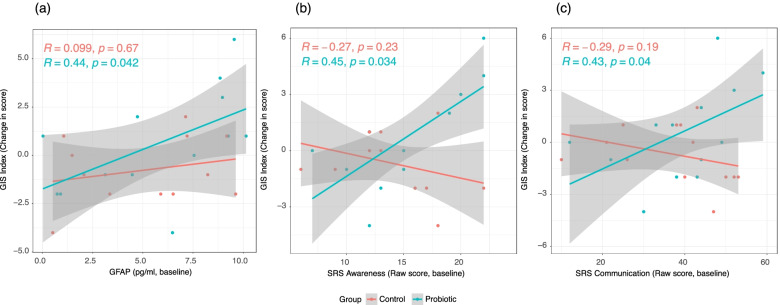
GSI change and its correlations with inflammatory marker and ASD severity

The change in GSI index among subjects receiving the active probiotic was found to be positively correlated with baseline GFAP concentration (Fig. 5A; *R*_*Probiotic*_ = 0.44, *P*_*Probiotic*_ < 0.05), SRS awareness (Fig. 5B; *R*_*Probiotic*_ = 0.45, *P*_*Probiotic*_ < 0.05), and SRS communication sub-score (Fig. 5C; *R*_*Probiotic*_ = 0.45, *P*_*Probiotic*_ < 0.05). However, these corresponding correlations are not found to be significant among the subjects receiving the placebo control (Fig. 5).

**Fig. 6:**
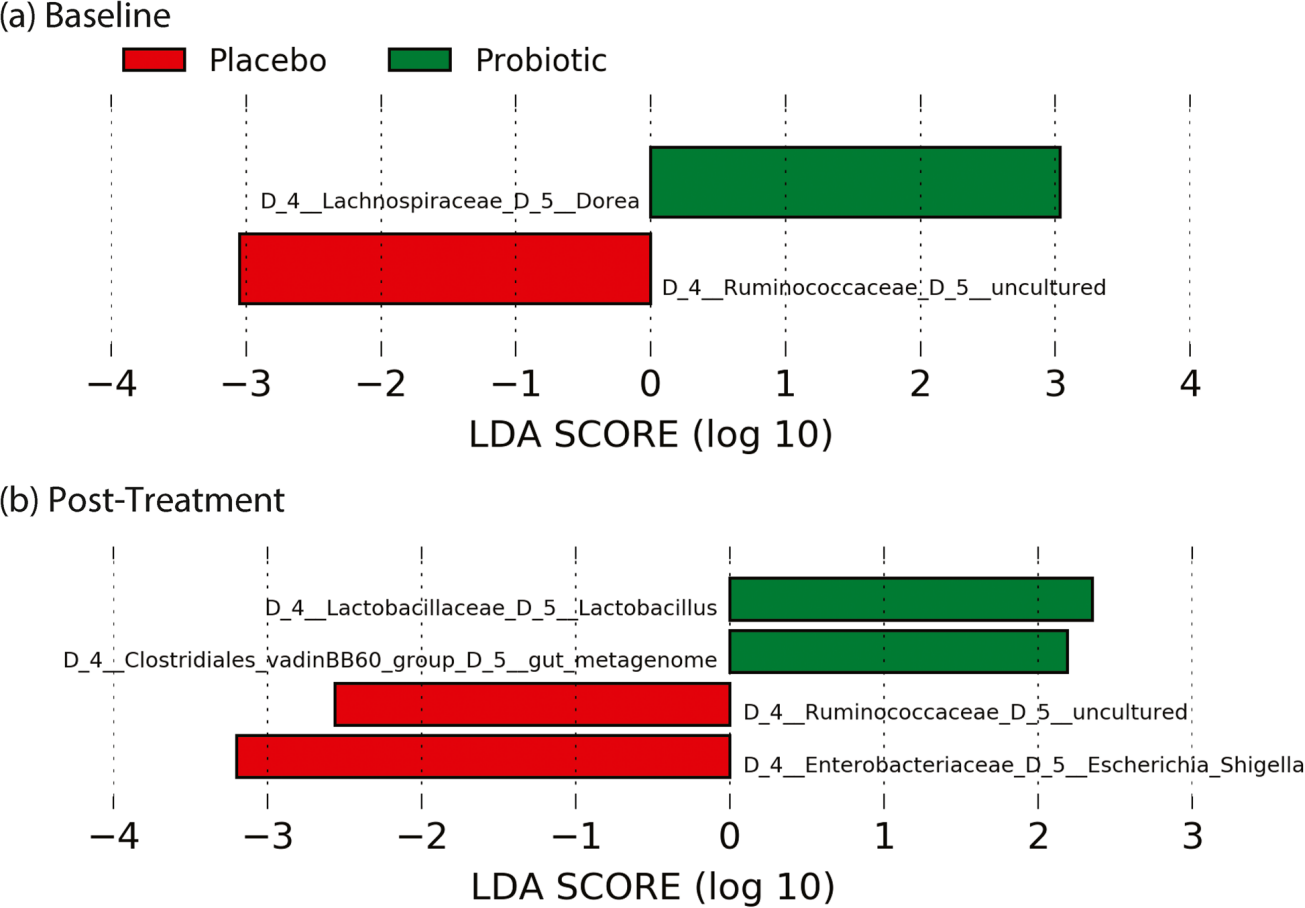
Differentially enriched microbiota between placebo and probiotic groups. Analyses was conducted at (**a**) baseline and (**b**) post-treatment for both groups via LEfSe

Assessment of differentially enriched microbiota by group both at baseline and post-intervention suggested several significant genus-level differences in relative abundance. The presented LDA scores of each microbiota with respect to the interventional groups were found to be significantly different at both baseline (Fig. 6A) and post-intervention (Fig. 6B) between probiotics interventional group and placebo control group (Fig. 6, *P* < 0.05).

**Fig. 7:**
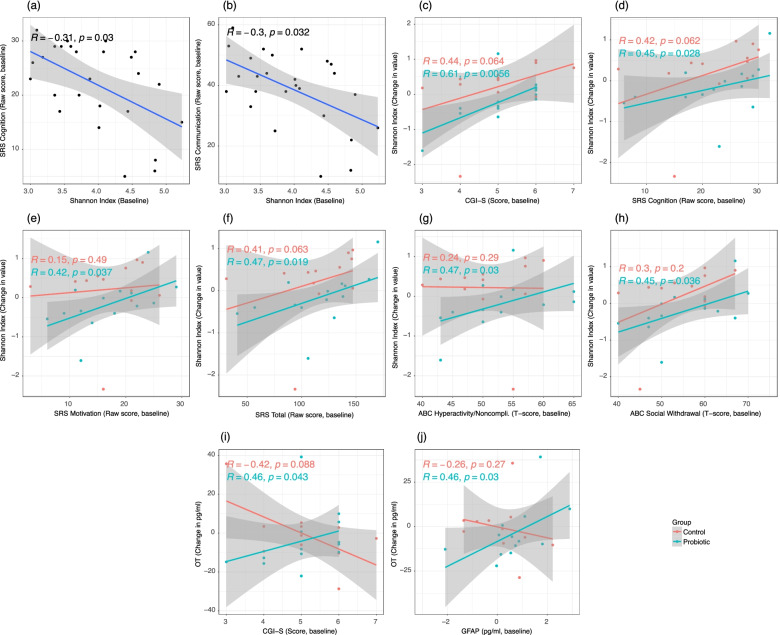
Correlations between point measurements and change in Shannon index, OT, GFAP, ASD severity, and autoantibody titers at both baseline and post-intervention

Baseline Shannon index was found to be negatively correlated with baseline scores of SRS cognition and SRS communication (Fig. 7A-B; *R* = -0.31, *P* < 0.05 and *R* = -0.3, *P* < 0.05, respectively). The change in Shannon index was found to be positively correlated with baseline CGI-S, SRS total score, SRS sub-scores, including cognition and motivation, and ABC-2 sub-scores, including hyperactivity/noncompliance and social withdrawal among subjects receiving the active probiotic (Fig. 7C-H, *P*_*Probiotic*_ < 0.05). The change in OT was found to be positively correlated with baseline measurements of CGI-S and GFAP among subjects receiving the active probiotic (*R* = 0.46, *P* < 0.05 and *R* = 0.46, *P* < 0.05, respectively) but these correlations are not observed among controls (Fig. 7I-J).

## Discussion

In the present study, we conducted a *post-hoc* exploratory analysis to examine and compare multiple inflammatory markers including SpCO measured by pulse CO-oximetry, several serum autoantibodies, cytokines, brain injury markers, OT level, gut microbiome composition, the correlations among them, and their correlations with ASD core symptom severity, GI symptom severity and the treatment response to the probiotic strain *L. plantarum* in randomized, double-blinded, placebo-controlled clinical trial. We found that positive measurements of inflammatory/autoimmunity markers were common among the study cohort but with varying prevalence: CaMKII and anti-tubulin are among the highest at a positive rate of 94.4%, followed by AECA with a rate of 82.6%, anti-D1 titer 77.8%, SpCO 64.7%, S100B 64%, anti-lysoganglioside GM1 and anti-D2L titer both 27.8%, IL-1β 24%, MBP 16%, which indicated that autoimmunity to tubulin, D1, AECA and related ongoing streptococcus infection are extremely common in this ASD cohort, autoantibodies to lysoganglioside GM1 and D2L are with much lower presence, while cytokine IL-1β and brain injury markers such as MBP are even less prevalent. From the previous Cunningham panel related studies indicated that D2L and tubulin correlated to ASD severity [5]. AECA was initially reported present in 30% of the ASD later reported significantly higher than control group and correlated with ASD severity [18, 19]. SpCO measured by pulse CO-oximetry was first explored in ASD population in this study, found to be abnormal in 64.7% of this ASD cohort, which is very close to inflammation rate found in the postmortem brains with multifocal perivascular lymphocytic cuffs contain increased numbers of lymphocytes in ~ 65% of ASD compared to control brains in males and females, across all ages, in most brain regions [50].

Interestingly, we first found that the SpCO level was significantly positively correlated with SRS total score at baseline, meaning SpCO could be indicative to ASD core symptom severity; Furthermore, the change of SpCO with intervention was found to be significantly positively correlated with serum GM1 level in probiotics treated group but not in placebo-treated group, which suggest those with lower level of GM1 had a greater improvement in the reduction of SpCO, which is hypothesized to be mediated through PS128 treatment. Similarly, the change in severity of ASD core symptoms by probiotics treatment were also found to be significantly positively correlated with GM1, and additionally with D1 and tubulin levels, these correlations were not found to be significantly associated among subjects of the placebo group. Taken together, these findings suggest that SpCO is a potentially promising autoimmunity/inflammatory biomarker for ASD. Considering the measurement of serum autoantibodies and inflammatory markers are requiring blood draw, not being able to perform in the local conventional labs, expensive price/mostly self-pay, SpCO measured by pulse CO-oximetry has a lot of advantage such as easy, quick (2-min), cheap, on-site, and completely non-invasive. We believe that SpCO could be promising to serve as a screening and diagnostic biomarker to indicate inflammation and autoimmunity therefore subgroup ASD for corresponding further testing or treatments, and also could be used for monitoring treatment outcome as we found in this study. As proposed, same as this promising application in ASD, SpCO could also be a useful quick test for COVID-19 to indicate inflammatory level, monitor severity, and clinical course, similarly for other conditions involved autoimmunity and inflammation [30]. The validation of the application in ASD, specific correlations with each condition and involved pathways are warrant for further studies.

Our previous study reported a strong correlation between serum level of cytokines and gut microbiome composition, others reported the serum level of autoantibodies are significantly correlated with ASD severity in both Cunningham panel and AECAs; however, the correlation between autoantibodies and gut microbiome and treatment response with probiotics were not explored in these previous studies [4, 5, 19]. In the present study cohort, the change in GSI showed positive associations against baseline SRS score and GFAP level (*P* < 0.05), suggesting that those with less severe ASD symptoms and lower GFAP levels show a greater extent of reduction in GI symptom severity, which are also potential better respondents to the administered probiotic. Moreover, the baseline gut microbiome alpha diversity was found to be negatively correlated with SRS cognition and communication sub-scores, which is consistent with our expectations. Additionally, the change in alpha diversity over the intervention course was found to be positively correlated with baseline measurements of CGI-S, ABC-2 and SRS, while being negatively correlated with baseline anti-D2L titer among the probiotic group subjects. These associations, however, are not observed with statistical significance among subjects receiving the placebo control, which likely indicates that the associations between changes in gut microbiome diversity, ASD core symptom severity, and autoantibodies are likely mediated through the supplementation of the probiotic. The abundance changes of certain microbiome with probiotics intervention support the inflammatory mechanism of ASD and anti-inflammatory effect of probiotics. Following intervention, subjects receiving the active probiotic displayed higher abundances of Lactobacillus as expected, and an unidentified genus belonging to the Clostridiales vadin BB60 group. Relative to the active probiotic group subjects, those receiving the placebo show higher abundances of an uncultured genus belonging to Ruminococcaceae and higher abundances of Escherichia-Shigella. While literature remains largely heterogenous regarding the clinical implications of the differentially abundant unidentified Clostridiales and Ruminococcaceae genera, there has been a wide body of literature supporting the anti-inflammatory effects through the production of short-chain fatty acids (SCFAs) by the increased Lactobacillus genus, both from our previous studies and external literature [51]. Furthermore, the observed higher abundances of Escherichia-Shigella within the gut microbiome in subjects receiving the placebo relative to that of the probiotic group suggests that the probiotic supplementation has the potential to suppress the dysbiosis of the Escherichia-Shigella genus, which is a well-known opportunistic pathogen and has been shown to be associated with higher severity in constipation among individuals with ASD [52]. These interesting findings of this secondary analysis study displayed the internal relationship of autoimmunity/inflammation, gut microbiome dysbiosis and ASD severity, and the therapeutic role of probiotics in this setting. As mentioned earlier, OT signaling may serve as a critical link in the gut-brain axis, OT was widely reported to have anti-inflammatory effects and therapeutic potential [38]. In this study, we found that the changes of OT was significantly positively correlated in the probiotic group with CGI-S and GFAP at baseline but not with placebo group subjects. OT was reported to be regulated by probiotics, and also interacted with other neurotransmitters and hormones [37, 53]. It’s warranted to further explore autoimmunity mediated inflammatory process and gut microbiome dysbiosis, the therapeutic role of probiotics and other anti-inflammatory/immunomodulatory treatments.

There are several limitations of the study that deserve consideration. 1) Despite our adoption of proper recruitment and retention strategies, the participant enrollment and retention for this trial were challenging. This is a secondary analysis study from the parent trial, the sample size is smaller due to limited availabilities of the test results and high dropout rate for blood draw; A small sample size limited the statistical power and further subgroup analysis. 2) The wide age range used in this study resulted in high subject population heterogeneity and potentially variable treatment efficacy. Future studies with a larger sample size and subgroup stratification are warranted. 3) Due to considerable Asian and other minority patients with some cultural and language barriers, in addition to multiple influencing factors on behavioral variabilities, the parent rating of social behavioral scales may be somewhat biased. 4) Adjustments for multiple comparisons was not performed as each correlational test was conducted in the presence of a pre-specified hypotheses. Nonetheless, we acknowledge this as a limitation as the abundance of correlational tests performed may introduce possibilities of false discovery. 5) Since this is a post hoc analysis of a previously conducted clinical trial of probiotics, there is a lack of comparison between enrolled individuals with ASD against healthy controls for serum biomarkers, which limited any direct comparisons. Despite the several limitations of our analysis strategy, we believe that the presented evidence in the current study is an efficient way to provide suggestive evidence that may serve as preliminary results for future studies in the elucidation of the interactions between ASD symptom severity and biomarkers of inflammation and autoimmunity.

## Conclusions

In the present secondary analysis of RCT study, we found a high rate of presence of serum anti-tubulin 94.4%, CaMKII 94.4%, AECA 82.6%, anti-D1 titer 77.8%, and SpCO 64.7% measured by pulse CO-oximetry in this ASD cohort, we first demonstrated that SpCO was positively correlated with ASD core symptom severity measured by total SRS score (*p* = 0.029), the improvement of SpCO by probiotics was found positively correlated with baseline GM1 level (*P* < 0.05) compared with placebo group. Similarly, ASD core symptom improvement with probiotics was also found to be positively correlated with GM1 and other autoantibodies. These findings indicated that easily administered, non-invasive SpCO a potentially promising autoimmunity and inflammatory biomarker to screen and subgroup ASD and monitor treatment response; The autoantibodies, gut microbiome profile, Serum OT level, GI symptom, and ASD corn symptom severity were all found to be highly correlated in probiotics treated group (*P* < 0.05) compared with placebo-treated group from this study, which warrant further studies to improve ASD early diagnosis and treatment outcome.

## Supplementary Information

**Additional file 1.** Overview of all assessed correlations.

## Data Availability

The data presented in this study are openly available in the Sequence Read Archive (SRA) database of The National Center for Biotechnology Information at https://www.ncbi.nlm.nih.gov/bioproject/PRJNA675093, with accession number PRJNA675093.

## Abbreviations

ASD: Autism Spectrum Disorder
SpCO: Carboxyhemoglobin
CFU: Colony Forming Unit
SRS: Social Responsiveness Scale
ABC-2: Aberrant Behavior Checklist, Second Edition
CGI: Clinical Global Impression
OT: Oxytocin
ELISA: Enzyme-linked Immunoassay
GI: Gastrointestinal
CDC: Centers for Disease Control
GFAP: Glial Fibrillary Acidic Protein
MBP: Myelin Basic Protein
S100B: S100 Calcium-binding Protein B
BBB: Blood–brain Barrier
AECA: Anti-endothelial Cell Antibodies
PANDAS: Pediatric Autoimmune Neuropsychiatric Disorders Associated with Streptococcal Infections
Anti-D1: Anti-dopamine Receptor D1
Anti-D2L: Anti-dopamine Receptor D2L
Anti-GM1: Anti-lysoganglioside GM1
CaMK II: Calcium Calmodulin-dependent Protein Kinase II
IVIg: Intravenous Immunoglobulin
HO-1: Heme Oxygenase-1
CO: Carbon Monoxide
COVID-19: Coronavirus Disease 2019
HC: Healthy Control
IL-1β: Interleukin-1β
*L. plantarum*: *Lactobacillus Plantarum*
IRB: Internal Review Board
SpO_2_: Oxygen Saturation
HR: Heart Rate
LEfSe: Linear Discriminant Analysis Effect Size
SRA: Sequence Read Archive
SCFA: Short-chain Fatty Acid
