# Supplementary material for: Carbon monoxide (CO) correlates with symptom severity, autoimmunity, and responses to probiotics treatment in a cohort of children with autism spectrum disorder (ASD): a post-hoc analysis of a randomized controlled trial
Source: BMC Psychiatry. 2022 Aug 8;22:536. doi: 10.1186/s12888-022-04151-3 (PMC9358122; doi:10.1186/s12888-022-04151-3)
Supplement: Supplementary file 1 — Additional file 1. Overview of all assessed correlations. [file 12888_2022_4151_MOESM1_ESM.docx]

**Additional file 1**. Overview of all assessed correlations.

|  | Baseline Cunningham Panel titers | Baseline SRS | Baseline ABC | Baseline CGI | Baseline GFAP |
| --- | --- | --- | --- | --- | --- |
| Baseline SpCO |  | X |  |  |  |
| Change in SpCO | X |  |  |  |  |
| Change in ASD severity (CGI/ABC/SRS) | X |  |  |  |  |
| Change in GSI |  | X |  |  | X |
| Baseline α diversity |  | X |  |  |  |
| Change in α diversity |  | X | X | X |  |
| Change in OT |  |  |  | X | X |
| Change in GFAP |  |  | X |  |  |

* Shaded area indicates all assessed correlations and “X” indicates a significant correlation identified within the present dataset.
